# The Risk of Tuberculosis Reinfection Soon after Cure of a First Disease Episode Is Extremely High in a Hyperendemic Community

**DOI:** 10.1371/journal.pone.0144487

**Published:** 2015-12-09

**Authors:** Pieter Uys, Hilmarie Brand, Robin Warren, Gian van der Spuy, Eileen G. Hoal, Paul D van Helden

**Affiliations:** 1 SACEMA, DST/NRF Centre of Excellence in Epidemiological Modelling and Analysis, University of Stellenbosch, Stellenbosch, Western Cape, South Africa; 2 DST/NRF Centre of Excellence for Biomedical Tuberculosis Research / MRC Centre for Molecular and Cellular Biology, Division of Molecular Biology and Human Genetics, Faculty of Health Sciences, University of Stellenbosch, Western Cape, South Africa; The Foundation for Medical Research, INDIA

## Abstract

Elevated rates of reinfection tuberculosis in various hyperendemic regions have been reported and, in particular, it has been shown that in a high-incidence setting near Cape Town, South Africa, the rate of reinfection tuberculosis (TB) disease after cure of a previous TB disease episode is about four times greater than the rate of first-time TB disease. It is not known whether this elevated rate is caused by a high reinfection rate due, for instance, to living circumstances, or a high rate of progress to disease specific to the patients, or both. In order to address that question we analysed an extensive data set from clinics attended by TB patients in the high-incidence setting near Cape Town, South Africa and found that, in fact, the (average) rate of reinfection (as opposed to the rate of reinfection disease) after cure of a previous TB disease episode is initially about 0.85 per annum. This rate diminishes rapidly over time and after about ten years this rate is similar to the rate of infection in the general population. Also, the rate of progress to disease after reinfection is initially high but declines in subsequent years down to the figure typical for the general population. These findings suggest that the first few months after cure of a TB disease episode form a critical period for controlling reinfection disease in a hyperendemic setting and that monitoring such cured patients could pre-empt a reinfection progressing to active disease.

## Introduction

It is known that in high TB incidence settings the rate of recurrent TB disease is much higher than the rate for first-time disease [1]. Many of these recurrent disease cases are due to relapse but the remaining cases are reinfection cases as confirmed by fingerprint comparison between the first episode and second episode. Indeed, it has been shown that, at least in one high incidence setting, the dominant cause of recurrent disease after the first year following cure is reinfection [2]. In the same setting the rate of reinfection disease appears to be as high as four times the rate of first-time infection (primary) disease [3]. High reinfection disease rates have also been reported from China [4] and elsewhere [1].

It is not clear why the rate of reinfection disease can be elevated compared to the rate of primary disease. It may be speculated that people who have already experienced a primary disease episode have just been unfortunate in finding themselves in a high risk socioeconomic environment possibly involving their workplace, school, club or transport situation, where other infectious cases continue to be present [5, 6]. Otherwise it could be that they are innately predisposed to progress to disease, possibly due to genetic risk factors [7, 8], lung damage or immunological deficiency caused by the previous episode, and perhaps smoking and drug abuse. It may be that a combination of these factors could be responsible. So a high rate of reinfection or a high rate of progress to disease or both could be responsible for the high rate of reinfection disease. The relative importance of these factors is the subject of much debate [9, 10] since direct observation of either of these rates is difficult. We estimated these rates by analysing an extensive data set collected from clinics serving a high TB-incidence setting near Cape Town, South Africa. The results of the statistical analysis were followed by a mathematical analysis that enabled the estimation of the average annual rate of reinfection and average annual rate of progress to disease corresponding to increasing time periods after cure. Our results show clearly that it is the very high average annual rate of reinfection immediately after cure that drives the high rate of reinfection disease. The rate of progress to disease is also elevated but to only a modest extent. We show that both these rates decrease over time.

## Methods

We estimate the annual rate of reinfection after cure of a previous first-time TB disease episode and also the rate of progression to active disease after reinfection has occurred using data from a particular hyperendemic community.

By ‘a reinfection disease episode’ we mean a disease episode resulting from a reinfection event occurring after cure of a previous disease episode as confirmed by the two episodes having different genotypes. By ‘cured’ we mean cured with bacteriological proof.

### Data

We used data for the period 1993 to 2005 from the epidemiological field site of Ravensmead/Uitsig, two adjacent hyperendemic urban communities of Cape Town.

These data had been collected and a subset of these data for the period 1993 to 1998 had previously been used by the authors GVDS, RW and PvH in the published paper 3 in the references. Another, overlapping, subset of these data for the period 1996 to 2008 had previously also been used by the authors GVDS and PvH in the published paper 2 in the references. These three different subsets were used as they represented the patient records that had been processed and which were available at the time the respective studies had commenced. All genotype work was done by these authors and placed in their own databank. None of the authors of this paper know the identity of the patients whose data was used. The patient information was anonymised by independent persons and these authors have no access to any information that can identify any person. The study was approved by SUN IRB (Stell Univ INst Review Board).

The main conclusions from these previous investigations were that:

1. The rate of reinfection TB disease was about four times that of new TB disease [3].
2. Overall, reinfection was the dominant cause of recurrent tuberculosis after treatment had been completed [2].

For the present investigation we considered it necessary to take the following into account:

1. It is possible that the rate of reinfection or the ensuing rate of progression to disease may vary depending on the number of previous TB disease episodes experienced by the patient so we considered only first reinfection disease episodes i.e. reinfection disease episodes after a cured first TB episode.
2. The rate of recurrent disease forms an upper bound for the rate of reinfection disease. A recurrent disease with the same strain as a previous disease episode may be the outcome of a reinfection event subsequent to the cure of the previous disease episode or it could just be a relapse of the earlier disease episode. Since it is not possible to distinguish between these two scenarios using the available data, we assumed conservatively, that a recurrent TB episode should be classified as a reinfection disease episode only if the genotypes for the two episodes were different–otherwise we assumed the second to be a relapse case. The reinfection rate estimated based on this assumption is likely an underestimate of the true reinfection rate since it is possible that patients could be reinfected with a commonly circulating strain or exposed to the same untreated index case.
3. We considered all patients who had documented first TB disease episodes that were cured with bacteriologic proof. For these patients we obtained an estimate of the time interval from the date of cure of the first disease episode to the date of the start of the recurrent TB disease episode. In the dataset only the dates on which a patient was seen at the clinic were documented. We therefore assumed that the date of cure is six months after commencement of treatment for susceptible TB and eighteen months for multiple drug resistant TB. We assumed that all TB cases for which resistance information was not sufficiently documented were not multiple drug-resistant i.e. had a treatment period of 6 months. It should be noted that since this assumption will result in an over-estimation of the time to reinfection disease for some cases, it will cause an underestimation of the rate of reinfection.
4. The estimated date of the start of the recurrent disease episode was determined based on the assumption that patients go to the clinic (and get diagnosed) as soon as symptoms appear, which is in turn assumed to occur one month after the disease episode actually begins. It may happen that a patient delays visiting a clinic so that his disease episode actually starts earlier than our estimate: If this was in fact the case for some patients, our assumption would contribute to under-estimation of the rate of reinfection disease.
5. Unfortunately fingerprint typing for both the first and the second disease episodes was not available for all the patients whose first episode had been cured (with bacteriological proof). It is reasonable to suppose that the cases where both episodes had been typed form a random sample of this larger population (i.e. genotyping information is missing completely at random). In reference [3] it is made clear that the patients with DNA fingerprints were representative for those without so that the cases where fingerprints were missing do not constitute a biased subset. We believe therefore that we may assume that fingerprints are missing in an entirely random way resulting from, for example, administrative error or loss of viability of specimens during transport from the clinics to the laboratories or just simple laboratory failures. So for those patients missing typing data, we assume the same proportion p_Diff_ experienced reinfection disease as in the subgroup with typing data available for both episodes. Since we believe this to be a reasonable assumption we will consider all cases with a cured first TB episode instead of only the subset with genotyping data available for both episodes. In this manner we obtain a larger subset to analyse. We noted that the proportion of the confirmed reinfection cases (where the first episode had been cured) compared to all recurrent cases (after cure) is *p*_*Diff*_ = 0.57. The risk of reinfection disease within a period of time t after cure of a first disease episode is then calculated as 0.57 times the risk of recurrent disease within a period of time t after cure of a first disease episode.

### Statistical Analysis

We considered all patients for whom there was a documented first TB disease episode that was cured with bacteriologic proof. For these patients we obtained an estimate of the time interval from the date of cure of the first disease episode to the date of infection with the recurrent TB infection. In the data set only the dates on which a patient was seen at the clinic were documented. So we estimated the date of infection for the recurrent episode on the assumption that patients go to the clinic (and get diagnosed) as soon as symptoms appear. This is assumed to occur 1 month after infection. Treatment for multi-drug resistant TB (defined as resistant to at least isoniazid and rifampicin) was assumed to be over an 18 month period while treatment of susceptible TB was assumed to take 6 months. The time interval was then calculated from the date of completion of the treatment until one month before diagnosis of the recurrent episode. This over-estimates the interval since it is most likely patients report to a clinic much later than one month after appearance of symptoms. Furthermore, again to be conservative, it was assumed that all TB disease cases for which resistance information was not documented (or sufficiently documented) were not multiple drug resistant and so had a treatment period of 6 months. For actual MDR cases this over-estimates the time to reinfection.

Survival analysis was performed on the time-to-event data. The survival function was estimated using the product-limit estimator [11]. In the analysis individuals who experienced recurrence after the cure of their first episode, were censored at the time of recurrence (meaning that they were only considered to be at risk of reinfection until that time and only contributed to the analysis until that time), while those who did not experience relapse or reinfection (as far as we know) prior to the 10^th^ of October 2005 (the date of the last clinic visit in the data set) were censored at this date. This type of censoring is called right censoring.

No information regarding for example migration or death was available after treatment ended for a patient. It was therefore necessary to make the assumption that if an individual did not return to the clinic, they survived recurrent TB disease as well as death. This assumption will most likely result yet again in an underestimate of the rate of reinfection. The survival analysis was performed using the R programming language and built in functions of an R package called ‘survival’. These functions correctly account for right censoring.

The pointwise confidence intervals for the Kaplan Meier curve for the survival of recurrent TB disease are derived from the confidence interval of the log hazard [12].

The confidence limits for the risk of reinfection disease can be obtained as follows. Since the risk of a recurrent TB disease episode within time t after cure is 1 minus the probability of surviving a recurrent TB disease episode for time t, the upper confidence limit of the risk of a recurrent disease episode within time t is 1 minus the lower confidence limit of the corresponding survival probability. Similarly for the lower confidence limit of the risk of a recurrent. The confidence limits for the risk of a reinfection disease episode (i.e. recurrent TB disease with a genotype different from that of the first) are *p*_*Diff*_ times the corresponding confidence limits of the risk of a recurrent TB disease episode.

It is this risk of reinfection disease over increasing time periods post cure of first disease episode that is examined in the next section, Mathematical Analysis, in order to investigate the annual rate of reinfection and the annual rate of progress to disease after reinfection.

### Mathematical Analysis

Here we present a summary of the method used to estimate the annual rate of reinfection and the annual rate of progress to disease.

We let *λ*_1_ and *λ*_2_ denote the annual rate of reinfection and the annual rate of progression to active disease after reinfection, respectively.

The probability, *D*, of becoming reinfected and then progressing to disease within *T* years after cure of a previous infection is given by

$$D=\int_{0}^{T}e^{-\lambda_{1}t}\lambda_{1}(1-e^{-\lambda_{2}(T-t)})dt$$

$$=-e^{-\lambda_{1}T}+\frac{\lambda_{1}}{\lambda_{1}-\lambda_{2}}{[e}^{-\lambda_{1}T}-e^{-\lambda_{2}T}]+1$$

$$=\frac{1}{\lambda_{1}-\lambda_{2}}{[\lambda_{2}e}^{-\lambda_{1}T}-\lambda_{1}\;e^{-\lambda_{2}T}]+1$$ (1)

**Fig 1:**
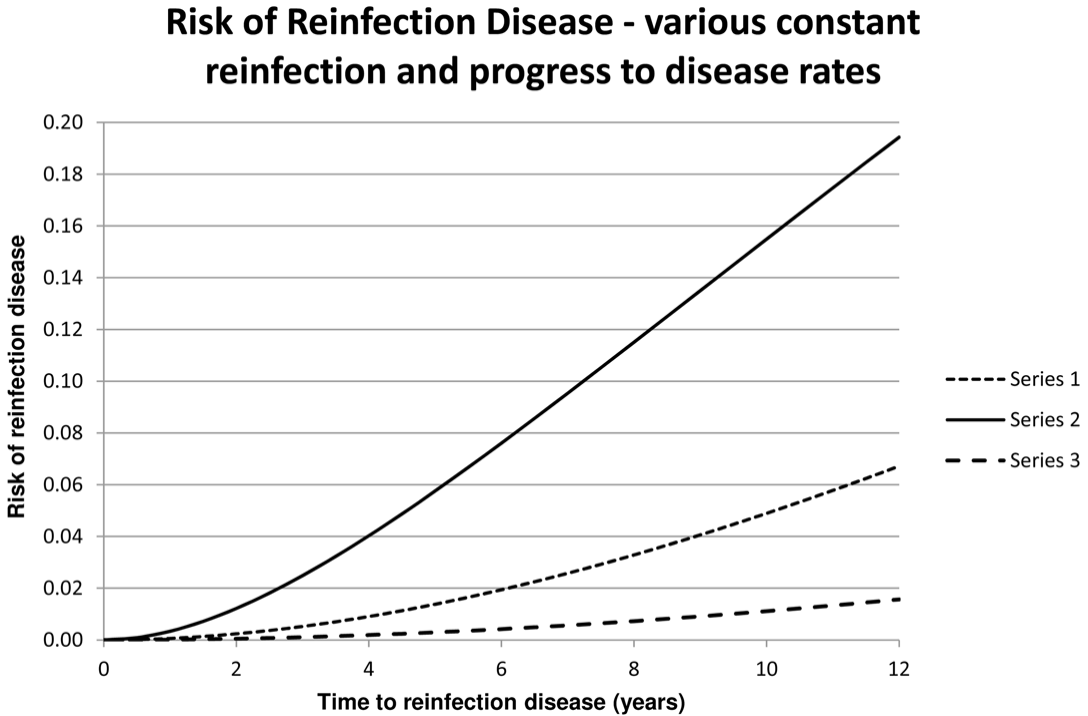
For Series 1 through 3 the values of *λ*_1_ and *λ*_2_ are respectively (0.05; 0.025), (0.3; 0.025) and (0.025; 0.01).

We observe that this shows that the probability, *D*, increases from 0 at time *T* = 0. Fig 1 shows *D* for some values of *λ*_1_ and *λ*_2_.

We now use the data regarding this probability to estimate *λ*_1_ and *λ*_2_.

We proceed from Eq (1) by calculating $\frac{dD}{dT}=\frac{{-\lambda }_{1}\lambda_{2}}{\lambda_{1}-\lambda_{2}}[e^{-\lambda_{1}T}-e^{-\lambda_{2}T}]$ and finding the second-order approximation (valid if *λ*_*i*_ *T* ≪ 1):

$$\frac{dD}{dT}\sim \;\lambda_{1}\lambda_{2}T\;[1-\frac{1}{2}(\lambda_{1}+\lambda_{2})T]$$ (2)

Let $R=\frac{\frac{dD}{dT}(T_{2})}{\frac{dD}{dT}(T_{1})}$. Then (2) shows

$$\lambda_{1}\lambda_{2}=\frac{\frac{dD}{dT}(T_{1})}{T_{1}\;[1-\frac{1}{2}(\lambda_{1}+\lambda_{2})T_{1}]}=a,\;\mathrm{say},$$

and $(\lambda_{1}+\lambda_{2})=\frac{2(RT_{1}-T_{2})}{\left[R{T_{1}}^{2}-{T_{2}}^{2}\right]}=b$, say.

Hence $\lambda_{i}=\frac{b\pm \sqrt{b^{2}-4a}}{2}$

It cannot be determined at this stage whether *λ*_1_ is given by $\frac{b+\sqrt{b^{2}-4a}}{2}$ or by $\frac{b-\sqrt{b^{2}-4a}}{2}$.

## Results

**Fig 2:**
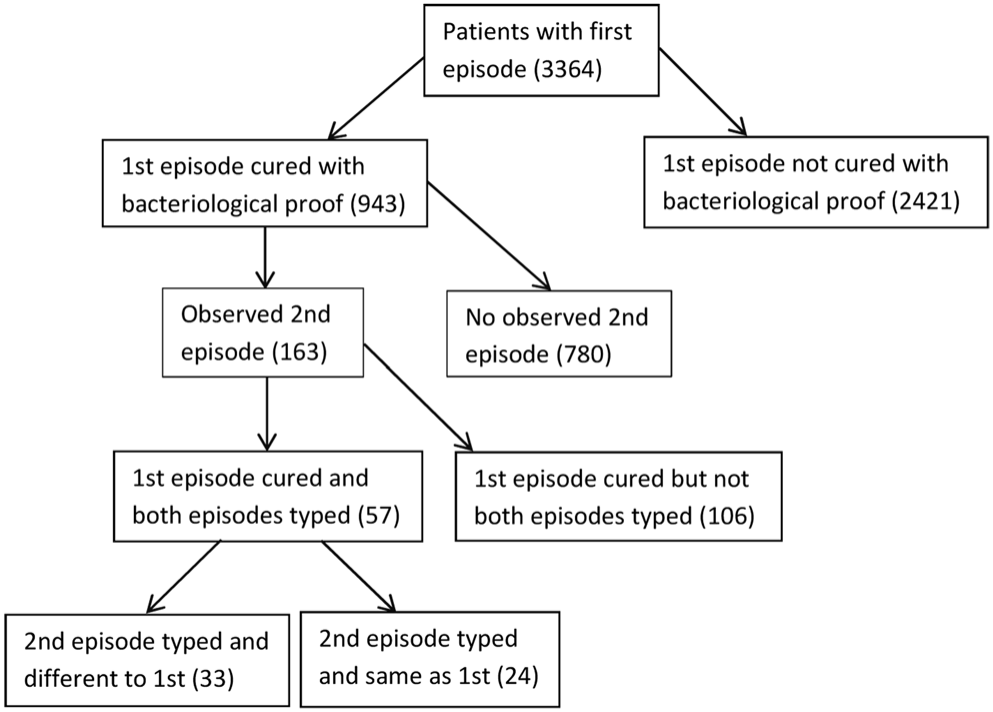
A schematic showing the breakdown of the 3364 patients with a documented first TB disease episode.

The five points made in the Methods section were taken into account when dealing with the data and the way these points determined which cases should be covered by our study (from the period 1993 to 2005) is best explained in Fig 2 which shows the number of patients in each of several categories of interest. Our analysis was applied to the 943 patients for whom the first disease episode was cured and the results are shown in Table 1.

**Table 1:** The risk of reinfection disease estimated from the data set.

Time since cure (days)	Number of patients at risk	Number of recurrent disease events	Survival Probability Recurrent Disease	Standard Error	CI for Survival of Recurrent disease: Lower Bound	CI for Survival of Recurrent disease: Upper Bound	Risk of Recurrent disease	Risk of Reinfection disease	CI for Risk of Recurrent disease: Lower Bound	CI for Risk of Recurrent disease: Upper Bound	CI for Risk of Reinfection disease: Lower Bound	CI for Risk of Reinfection disease: Upper Bound
0	943	0	1	0	1	1	0	0	0	0	0	0
182	895	23	0.975392	0.005068	0.9655	0.9854	0.024608	0.014027	0.0146	0.0345	0.0083	0.0197
365	829	18	0.955216	0.00684	0.9419	0.9687	0.044784	0.025527	0.0313	0.0581	0.0178	0.0331
548	752	14	0.938316	0.008075	0.9226	0.9543	0.061684	0.03516	0.0457	0.0774	0.0261	0.0441
730	697	11	0.924319	0.00899	0.9069	0.9421	0.075681	0.043138	0.0579	0.0931	0.033	0.0531
912	609	18	0.89878	0.010569	0.8783	0.9197	0.10122	0.057695	0.0803	0.1217	0.0458	0.0694
1095	564	11	0.882187	0.011497	0.8599	0.905	0.117813	0.067153	0.095	0.1401	0.0541	0.0798
1278	518	9	0.867599	0.012293	0.8438	0.892	0.132401	0.075469	0.108	0.1562	0.0615	0.089
1460	474	7	0.855486	0.012947	0.8305	0.8812	0.144514	0.082373	0.1188	0.1695	0.0677	0.0966
1642	440	9	0.838749	0.013844	0.8121	0.8663	0.161251	0.091913	0.1337	0.188	0.0762	0.1071
1825	408	7	0.824892	0.014572	0.7968	0.854	0.175108	0.099812	0.146	0.2032	0.0832	0.1158
2008	382	6	0.812487	0.015208	0.7832	0.8428	0.187513	0.106882	0.1572	0.2168	0.0896	0.1236
2190	345	9	0.792676	0.016209	0.7615	0.8251	0.207324	0.118175	0.1749	0.2385	0.0997	0.1359
2372	312	2	0.787879	0.016462	0.7563	0.8208	0.212121	0.120909	0.1792	0.2437	0.1021	0.1389
2555	284	3	0.779894	0.016928	0.7474	0.8138	0.220106	0.12546	0.1862	0.2526	0.1061	0.144
2738	257	2	0.774154	0.017284	0.741	0.8088	0.225846	0.128732	0.1912	0.259	0.109	0.1476
2920	230	4	0.761496	0.018123	0.7268	0.7979	0.238504	0.135947	0.2021	0.2732	0.1152	0.1557
3102	206	3	0.751048	0.018852	0.715	0.7889	0.248952	0.141903	0.2111	0.285	0.1203	0.1625
3285	186	1	0.74733	0.019122	0.7108	0.7858	0.25267	0.144022	0.2142	0.2892	0.1221	0.1649
3468	146	1	0.742381	0.019625	0.7049	0.7819	0.257619	0.146843	0.2181	0.2951	0.1243	0.1682
3650	111	0	0.742381	0.019625	0.7049	0.7819	0.257619	0.146843	0.2181	0.2951	0.1243	0.1682
3832	72	1	0.734648	0.020889	0.6948	0.7768	0.265352	0.151251	0.2232	0.3052	0.1273	0.1739
4015	48	1	0.720243	0.024957	0.673	0.7709	0.279757	0.159461	0.2291	0.327	0.1306	0.1864
4198	23	0	0.720243	0.024957	0.673	0.7709	0.279757	0.159461	0.2291	0.327	0.1306	0.1864
4380	9	0	0.720243	0.024957	0.673	0.7709	0.279757	0.159461	0.2291	0.327	0.1306	0.1864

For the data covered by our analysis, *p*_*Diff*_ (which was defined in the Methods section) is equal to 0.57 (33/57—Table 1). We can therefore expect that about 93 of the 163 patients (0.57*163 = 93) with a cured first episode and an observed second episode (see Fig 2) experienced that second episode as a result of reinfection. This represents about 10% of the 943 patients with a cured first disease episode. Thus reinfection disease constitutes a significant proportion of the case load.

**Fig 3:**
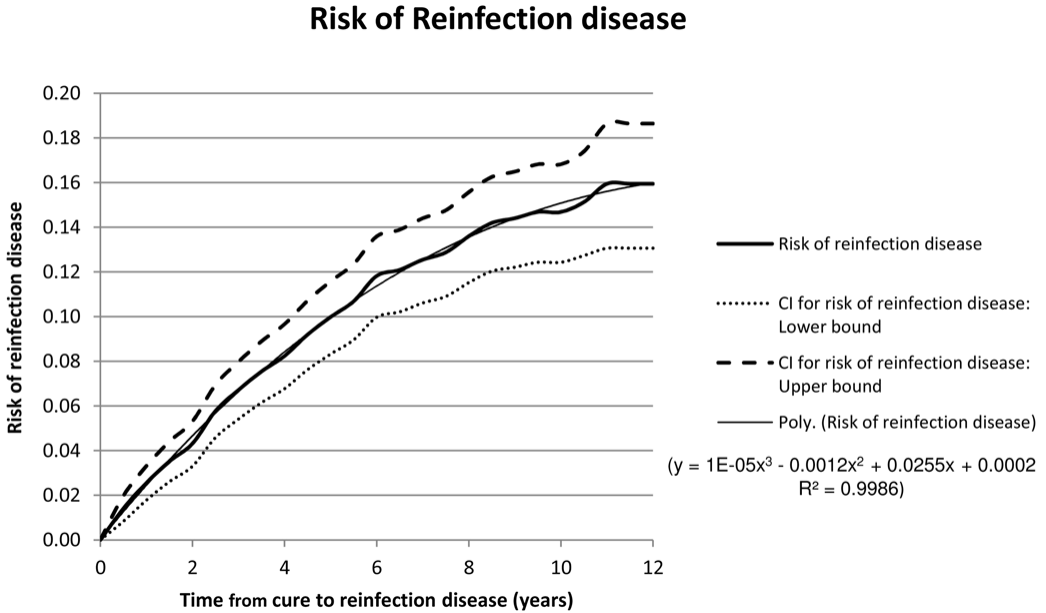
The mean risk of reinfection disease manifesting a given number of years after cure of a prior cured disease episode is shown together with confidence intervals. Trend lines are fitted and these are used to estimate the gradients of the risk graphs. The trendline for the mean risk of reinfection disease only is shown and its parameters are displayed.

The risk of reinfection disease as determined from the data and recorded in Table 1 increases as a function of time from cure of the first disease episode and is shown graphically in Fig 3.

**Fig 4:**
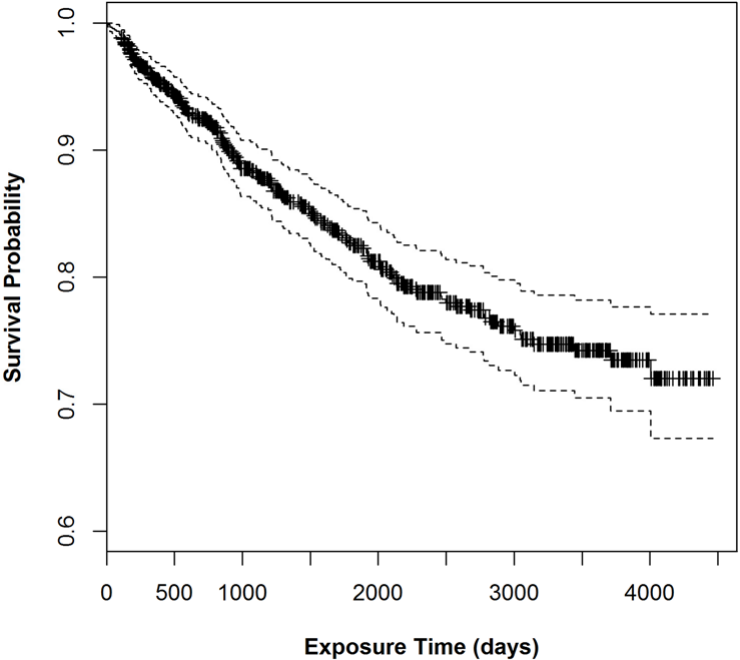
The Kaplan Meyer survival plot from Table 1.

The Kaplan Meyer survival plot is shown in Fig 4.

Comparison of the graphs in Fig 3 with the theoretical graphs in Fig 1 shows that the rate of reinfection and the rate of progress to disease cannot be constant. Rather, it appears that in fact the rates are high initially and decrease during the subsequent years.

In Fig 3 a polynomial trend line is fitted to the graph of the probability of reinfection disease. This polynomial is differentiated to obtain the gradient to the probability graph for various times after cure. This is done for the Upper and Lower confidence interval bounds as well.

**Fig 5:**
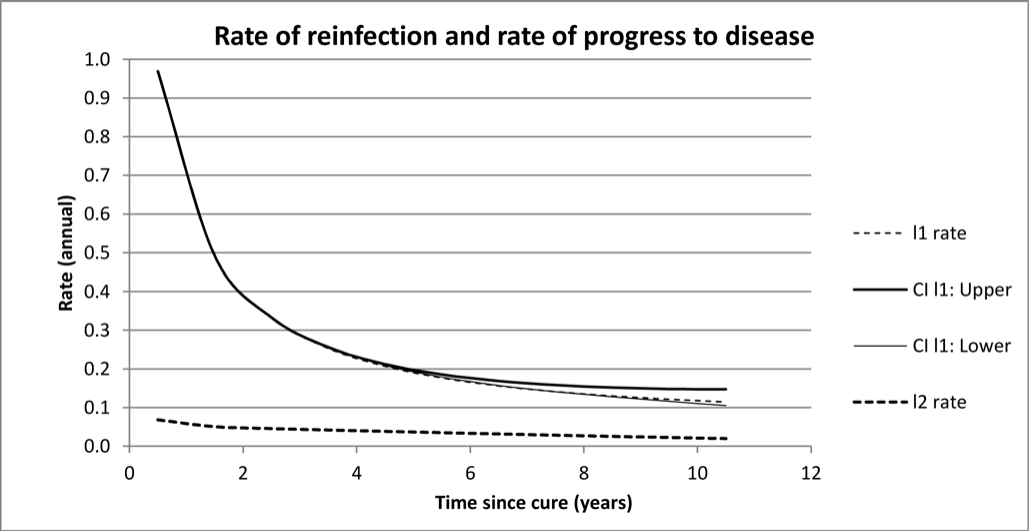
The confidence intervals for l_2_ are not plotted since they are too small to be visible.

This enables the estimation, using the formulae in Methods, of quantities l_1_ and l_2_ (Table 2), these being candidates for *λ*_1_ and *λ*_2_. (Recall that the formulae derived in the Methods section do not distinguish between the rate of reinfection and the rate of progress to disease). l_1_ and l_2_ are plotted in Fig 5. Fig 5 shows that l_1_ and l_2_ decrease over time with l_2_ tending towards a value typical for the generally accepted value of the rate of progress to disease. We deduce then that l_1_ represents the annual rate of reinfection after cure i.e. *λ*_1_.

**Table 2:** Rate of reinfection disease, with confidence intervals for various times after cure.

	CI Upper		Rate		CI Lower	
Time	l1	l2	l1	l2	l1	l2
0.5	0.962	0.079	0.966	0.068	0.969	0.057
1.5	0.494	0.058	0.493	0.051	0.492	0.043
2.5	0.334	0.051	0.333	0.046	0.333	0.040
3.5	0.256	0.046	0.253	0.042	0.256	0.037
4.5	0.209	0.041	0.207	0.038	0.212	0.034
5.5	0.179	0.037	0.177	0.035	0.186	0.031
6.5	0.157	0.033	0.156	0.032	0.169	0.028
7.5	0.141	0.029	0.141	0.029	0.158	0.025
8.5	0.128	0.026	0.130	0.025	0.152	0.022
9.5	0.116	0.022	0.121	0.023	0.148	0.019
10.5	0.105	0.019	0.114	0.020	0.147	0.017

Recall that the derivation of the formulae assumes that *λ*_1_ and *λ*_2_ are constant over time. Application of these formulae will therefore yield estimates for average values of *λ*_1_ and *λ*_2_ over the selected time periods. In particular, the values found for larger times *T* will be inflated because they are affected by contributions from earlier years when the values are high. Despite these limitations, Fig 5 shows that initially the values are very high with the average value of *λ*_1_ about 0.85 during the first year after cure. These values drop off very quickly. The size of the estimate of the average annual rate of reinfection over 10 years after cure (0.114 per annum) suggests that after about 10 years the rate of reinfection is similar to the rate of (first) infection for the population in general.

## Conclusion

It has been established elsewhere that in hyperendemic settings reinfection is the major cause of recurrent TB disease. We found in the present study pertaining to the specific settings identified that it can be expected that approximately 57% of patients who were cured of a first episode and who experienced a recurrent episode, experienced reinfection as opposed to relapse.

These reinfection cases constitute a significant proportion (at least 10%) of all cases where the first episode had been cured. Moreover we also note that 43% of the patients who were cured with proof had a second episode with the same strain as the first. For the purposes of our analysis we conservatively regarded these as relapse cases but it is quite likely that such patients had been reinfected by commonly circulating strains or even by the (untreated) people responsible for infecting them the first time. In fact, we have previously found an association of specific Mtb strains with humans with a particular HLA type [13], and this could also explain why, in a hyperendemic setting with a number of strains present in the environment, the strain infecting the host the second time is often the same as the first, as this strain is the best “fit” for that host. Since reinfection with the same strain was an exclusion criterion here, the estimated rate of reinfection leading to disease is an underestimate.

Our study revealed that patients cured of a first disease episode were at great risk of becoming reinfected during the initial months after cure. Initially the annual reinfection rate is about 0.85, (see the Appendix for a very simple explanation of why this is actually a perfectly reasonable value for the reinfection rate) but the rate drops off very rapidly during the subsequent two to three years and continues to drop so that by about ten years since cure the rate has a value typical of that for the population generally. It may be hypothesised that these patients continued in a high risk environment after cure but such high risk conditions diminish over time for them as, for example, they move to different employment or the infectious people to whom they had been exposed become cured themselves. But we also know that human genetic susceptibility plays a significant role in determining which of the latently or newly infected hosts progress to active disease [14], and we may expect these hosts to have repeated episodes of disease in a hyperendemic area. We hypothesize that different allelic frequencies in genes that are crucial at a number of stages in the immune defence against Mtb will impact the host resistance to this infection and its progression. Within an admixed population such as that investigated here, there will be a wide variety of susceptibility alleles, some derived from different ancestral input [15, 16, 17] and others representing normal variation in any population [18, 19, 20, 21]. Resistance to TB may encompass resistance to infection, or resistance to progression of disease, and our linkage studies in this community show that two separate loci are involved [22], and we postulated that patients who maintain a zero measure for skin test positivity, are inherently resistant. This cohort would by definition not have the first episode of TB, and would therefore not be included in the present study. Those who are most susceptible will succumb rapidly again after the first infection, while as time passes and this susceptible cohort is “removed” from the pool of patients recovering from TB, the apparent rate of reinfection will go down. This mechanism is supported by Rodrigues et al [23] and Gomes et al, [24], as fitting the data better than the hypothesis that infection increases susceptibility to reinfection.

However, we need to consider another possible explanation for the high rate of reinfection shortly after cure; namely, that the patient’s immune system remains compromised for a significant period after bacterial cure, as a result of the disease episode. Cytokine levels and networks may remain disrupted due to their slow recovery after an episode of TB. In patients cured of an episode of TB, the levels of IFN-γ immunoreactivity remain suppressed at the end of therapy [25] and for at least 12 months after the start of therapy [26]. IFN-γ is important in the TH1-type cytokine response, and while a depressed level of IFN-γ activity may point to genetic susceptibility in some patients, the levels at 18 months have recovered to control levels, indicating the time dependence of the effect [26]. The ratios of other cytokine such as TNF-α/IL-10 were significantly increased in TB patients before, during, and also at the end of treatment compared to those of control subjects [25]. Pathogenic mycobacteria can subvert the autophagy/apoptotic pathways, and a number of signalling cascades, and the extent and duration of these effects is not known.

The delicate balance between the survival of the pathogen and the success of the host defence system could be tipped in favour of disease success in the months after an infection, for a combination of all the above reasons [27] and this, in combination with the genetic susceptibility previously discussed, manifests as an early increase in disease after cure.

The first episode of TB has already served to identify those infected persons that are likely to progress to disease, and the public health benefit of monitoring this subset of the population for a short period to prevent the even smaller subset which may be the most genetically susceptible and is at increased risk of a second episode of disease, seems well worth the effort.

Whether such monitoring should be implemented and for how long such monitoring should continue would be best determined by a cost-benefit analysis compared to other types of control measures.

This study does not provide definitive answers as to why the rate of actual reinfection soon after cure is so high. However, should monitoring take place, it is likely that the reason a patient has become reinfected so quickly could become evident. This in itself could suggest more efficient measures.

## Supporting Information

S1 Appendix (DOCX)
