# Supplementary material for: The Risk of Tuberculosis Reinfection Soon after Cure of a First Disease Episode Is Extremely High in a Hyperendemic Community
Source: PLoS One. 2015 Dec 9;10(12):e0144487. doi: 10.1371/journal.pone.0144487 (PMC4674135; doi:10.1371/journal.pone.0144487)
Supplement: S1 Appendix — (DOCX) [file pone.0144487.s001.docx]

**Appendix**

**Simple explanation showing high reinfection rates are plausible**

Reference 2 concludes that ‘The reinfection disease rate after successful treatment was estimated at 2.2 per 100 person-years’. Thus, accordingly, 22 people out of 1000 could have active reinfection disease within one year after cure. Our finding that the reinfection rate after cure is initially 0.85 per year indicates that after six months approximately 425 (850/2) of a cohort of such 1000 people would be reinfected. Assuming the usual rate of progress to active disease of 5% per annum, during the remaining 6 months of that year the number of the reinfected people progressing to active disease would be ½ of 5% of 425 i.e. 10.6. In the same way the number of people becoming reinfected and progressing to disease during the second half of the year could be estimated. The estimate would be somewhat less than 10.6. So based on a reinfection rate of 0.85 we could expect about 21 (2 x 10.6) people out of a cohort of 1000 to develop reinfection disease during the first year after cure of a first disease episode.

**Reason for the discrepancy between our findings in our Fig 4 and the Fig 2 in reference 3**

Verver et al [3] presented (in figure 2) a Kaplan Meier analysis from the same highly burdened setting that showed that the proportion of patients without recurrence is around 95% at 6 years after successful treatment of an initial episode (i.e. 5% got recurrence at 6 years). The current paper shows a six-year rate of reinfection from the same community (in figure 3) to be 12% or double that value at six years. The reason for this difference is as follows:

Verver et al [3] consider a recurrence to be a reinfection only if it is confirmed by fingerprint - they ignored the other recurrences for which there was no fingerprint.

They considered 358 patients who were cured of which 48 in total experienced recurrence in the approximately 5-year period 1993 to 1998. However, only 24 of the 48 had DNA fingerprints that could be used to confirm reinfection. Reinfection was confirmed for 19 of the 24 patients with fingerprints and hence the risk of reinfection disease reinfection over the approximately 5-year period was found to be 19/358 = 5.3%.

But if one assumes that fingerprints are missing completely at random then we can expect that (19/24)*48 = 38 of the 48 patients had reinfection which would put the total risk of reinfection over the approximately 5-year period at 38/358 = 10.6%. This is double the estimate given by Verver et al and is similar to the figure we find if we focus on the period 1993 to the end of 1998 and exclude MDR cases (actually excluding the MDR cases does not make that big a difference). The 10.6% is close to what we found in our original results (which considers 1993 - 2005 and includes MDR cases).
